# Tamsulosin and Solifenacin in the Treatment of Benign Prostatic Hyperplasia in combination with overactive bladder

**DOI:** 10.12669/pjms.334.12757

**Published:** 2017

**Authors:** Hui Wang, Yanhua Chang, Hui Liang

**Affiliations:** 1 Hui Wang, Department of Urology Surgery, Binzhou People’s Hospital, Shandong 256610, China; 2 Yanhua Chang, Department of Urology Surgery, Binzhou People’s Hospital, Shandong 256610, China; 3 Hui Liang, Disinfection Supply Room, Binzhou City Center Hospital, Shandong, 251700, China

**Keywords:** Overactive bladder, Solifenacin, Tamsulosin

## Abstract

**Objective:**

To analyze the clinical effect of tamsulosin and Solifenacin in the treatment of benign prostatic hyperplasia in combination with overactive bladder and its safety. Another objective was to investigate the clinical effect and safety of mega dose of tamsulosin in the treatment of benign prostatic hyperplasia in combination with overactive bladder.

**Methods:**

One hundred and twenty-four patients who were admitted to the Dept. of Urology at Binzhou People’s Hospital, , China with confirmed benign prostatic hyperplasia (BPH) with overactive bladder were randomly divided into two groups. Sixty-two patients in the control group were treated with tamsulosin, while sixty-two patients in the observation group were treated with tamsulosin in combination with solifenacin. The treatment of both groups lasted for 12 weeks. The effect and adverse reaction were compared between the two groups.

**Results:**

The international prostate symptom score (IPSS), quality of life (QOL), and overactive bladder symptom score (OABSS), Q_max_, pulmonary vascular resistance (PVR), daytime urination frequency, urgent urination frequency, urge urinary incontinence frequency and night urinary frequency of both groups improved after treatment, and the difference had statistical significance (P<0.05). The differences of the observation indexes (except PVR) in the observation group before and after treatment was significantly different with those of the control group (P<0.05). The incidence of adverse reactions in the observation group was lower than that in the control group, but the difference had no statistical significance (X^2^=2.843, P>0.05).

**Conclusion:**

Treating benign prostatic hyperplasia in combination with overactive bladder with tamsulosin in combination with solifenacin is more effective than tamsulosin, without significantly increasing adverse reactions. Thus the therapy is worth clinical promotion.

## INTRODUCTION

Bladder outlet obstruction (BOO) caused by benign prostatic hyperplasia (BPH) is often accompanied with overactive bladder. Knutson et al. found that fifty percent of patients with BOO had overactive bladder (OAB).^1^ OAB, a clinical syndrome caused by abnormal afferent signals and detrusor overactivity, used to be called unstable bladder, the main symptoms of which were urgent urination, uroclepsia, frequent micturition, increased urination at night, and it would seriously influence patients’ life quality.^2^,^3^ Study showed that the more obvious prostatic median lobe hyperplasia was and more serious BOO symptoms were,^4^ the severer OAB symptoms were; the reason why OAB occurred was secondary changes of stability, elasticity and sensitivity of bladder which was caused by BOO. For patients with BPH and OAB, the key of medication treatment was increasing urinary bladder pressure and contraction of bladder to promote urination, inhibiting bladder contraction, reducing afferent sensation to improve storage symptoms. Clinically, ±-receptor blocker and M receptor antagonist are the preferred medicines for lower urinary tract symptoms induced by BOO and BPH.^5^-^7^ However, the improvement of storage symptoms is not ideal for some patients when they are treated with ±-receptor blocker because the symptoms are induced by detrusor over activity rather than BPH.

In recent years, α-receptor blocker in combination with M receptor antagonist has been found more effective than single medication in treating urinary tract syndrome.^8^ Therefore, further analyzing the effect of α-receptor blocker in combination with M receptor antagonist in the treatment of BPH and OAB is of great clinical significance..

## METHODS

### Research Objects

One hundred and twenty-four patients with BPH in combination with OAB who were admitted to department of urology of our hospital between April 2014 and December 2015 were selected as the research subjects. Patients who were confirmed as prostatic hyperplasia by B ultrasound, computed tomography (CT), detection of prostate-specific antigen (PSA) and rectal touch, had typical lower urinary tract symptoms, and had international prostate symptom score (IPSS) no lower than 8 points, overactive bladder symptom score (OABSS) higher than 3 points (score of urgent urination higher than 2 points), average urination frequency no less than 8 times/d, frequency of night urination no less than 2 times and urinary volume no less than 200 mL were included. Those who had neurogenic diseases affecting bladder function such as spinal cord injury, prostatic cancer and urethrostenosis, severe lower urinary tract obstruction (residual urine volume no less than 200 mL) or acute infection, underwent urethra previously, or took other α- or M-receptor blocker within two weeks were excluded. The 124 patients were randomly divided into a control group and an observation group, 62 in each group. This study has been approved by the ethics committee of the hospital, and all the included patients signed informed consent.

The patients orally took placebo for four weeks at first and then treated for 12 weeks. Patients in the control group orally took 0.2 mg of tamsulosin after breakfast, once each day, for 12 weeks. Patients in the observation group orally took 0.2 mg of tamsulosin and 5 mg of solifenacin after breakfast, once a day, for 12 weeks.

The patients were evaluated on the grouping day and at the end of the treatment respectively. Observation indexes included international prostate symptom score (IPSS), quality of life (QOL), and overactive bladder symptom score (OABSS), Qmax, Post Void Residue (PVR), daytime urination frequency, urgent urination frequency, urge urinary incontinence frequency and night urinary frequency. They were followed up for two weeks after treatment, and the improvement of symptoms and the incidence of adverse reactions were recorded.

### Statistical Analysis

Data were analyzed by SPSS ver. 20.0. Measurement data were expressed as mean±SD, comparison within groups was performed using sample t test and comparison between groups adopted pair t-test; enumeration data were processed by Chi-square test, and P<0.05 meant that difference was statistically significant.

## RESULTS

The differences in the age, quality of prostate, IPSS, QOL, Q_max_ and PVR were not statistically significant (P>0.05) (Table-I).

**Table-I:** Comparison of general data between two groups (mean±SD).

*Groups*	*Observation group(N=62)*	*Control group(N=62)*	*t*	*P*
Ages/years old	65.2±4.7	65.4±5.1	0.468	>0.05
Weight of prostate/g	43.6±7.8	44.7±9.2	1.879	>0.05
PVR/mL	38.7±14.3	35.1±13.8	0.487	>0.05
Qmax/mL·s^-1^	8.4±2.2	8.5±1.9	0.593	>0.05
QOL/scores	3.9±1.1	3.8±1.0	0.527	>0.05
IPSS/scores	18.2±2.3	18.5±2.1	0.396	>0.05

### Curative effects of patients in the two groups

The subjective observation indexes such as IPSS, OABSS and QOL of the observation group and the control group were improved after treatment, and comparison within groups suggested significant difference (P<0.05). The differences of indexes in the observation group before and after treatment were significantly different with those in the control group before and after treatment (P<0.05). The objective indexes such as PVR, Q_max_, daytime urination frequency, night urination frequency, urge urinary incontinence frequency and urgent urination frequency of both groups improved after treatment, and the difference within groups had statistical significance (P<0.05). The difference of Q_max_, daytime urination frequency, night urination frequency, urge urinary incontinence and urgent urination before and after treatment in the observation group was significantly different with that in the control group (P<0.05). The difference of PVR before and after treatment in the two groups was remarkably different (P>0.05; Table-II).

**Table-II:** Comparison of different indexes before and after treatment between the two groups before and after treatment.

*Index*	*Observation group*			*Control group*			*t*	*P*

	*Before*	*After*	*Difference*	*Before*	*After*	*Difference*		
IPSS/point	18.2±2.3	9.8±2.7	8.5±2.7	18.5±2.1	12.8±2.7	5.3±4.5	5.549	<0.05
OABSS/point	8.4±2.2	4.8±1.2	3.6±1.4	8.5±1.9	6.2±1.8	2.3±0.7	4.402	<0.05
QOL/point	3.9±1.1	1.8±0.6	2.1±0.7	3.8±1.0	2.3±0.8	1.5±0.4	3.113	<0.05
Daytime urination/times	13.3±3.4	7.8±2.1	5.5±2.0	12.9±3.8	8.9±3.3	4.1±2.5	2.834	<0.05
Night urination/times	3.3±1.4	1.2±0.5	2.1±1.1	3.8±1.7	2.6±1.1	1.2±0.9	3.625	<0.05
Urge urinary incontinence/times	2.1±1.6	1.0±1.1	1.1±1.0	1.9±1.4	1.4±1.0	0.5±0.8	2.174	<0.05
Urgent urination/times	6.6±1.6	3.1±1.4	3.4±1.6	6.5±1.4	4.3±1.6	2.2±1.1	3.589	<0.05
PVR/mL	38.7±14.3	19.1±9.0	19.6±11.7	35.1±13.8	20.6±9.4	15.3±10.3	1.598	>0.05
Q_max_/mL·s^-1^	8.4±2.2	11.9±3.0	3.5±2.1	8.5±1.9	10.8±2.3	2.3±1.5	2.224	<0.05

### Incidence of Adverse Reactions

There was no acute urinary retention in both groups. The incidence of adverse reactions in the control group was 17.74% (11/62), among which dizziness was 3.23% (2/62), thirst was 4.84% (3/62), blurred vision was 6.45% (4/62), and aggravated dysuria was 3.23% (2/62). The adverse reaction incidence of the observation group was 11.29% (7/62), among which dizziness was 3.23% (2/62), thirst was 4.84% (3/62), blurred vision was 3.23% (2/62), and aggravated dysuria was 3.23% (2/62). The difference in adverse reaction incidence between the two groups was not statistically significant (P>0.05).

## DISCUSSION

BPH is the common cause of LUTS of middle-aged and old males, and LUTS in storage period can produce more obvious influence on living quality.^9^ OAB is characterized by urgent urination, frequent micturition and urge urinary incontinence, which is similar to LUTS in storage period. Almost 50% of BPH patients have OAB. Its cause has not been clarified clearly, and it is assumed to be associated to bladder mucosa, myogenic factors or neurogenic factors.^10^ α-receptor blocker is the preferred drug for the treatment of BPH induced LUTS. Tamsulosin as a novel drug, can significantly inhibit sympathetic nerve α1 receptor, selectively block α_1A_ receptor, effectively act on the urethra, bladder and prostate, and highly selectively block smooth muscle. Its effect on the symptoms of storage period is not good as expected though its inhibitory effect on the increase of urethral pressure is 13 times that of the inhibitory effect on the increase of vascular diastolic pressure.^11^

M-receptor blocker mainly works in storage period. It can inhibit the involuntary shrink of detrusor through inhibiting nervous impulse. The effectiveness of M-receptor blocker in combination with α-receptor blocker has been verified by many studies. Kaplan SA et al.^12^ selected 95 BPH patients who aged no less than 40 years old and had 12 points more IPSS, 3 points more QOL, no less than 8 times of urination each day and no less than 3 times of urination each day and divided them into a control group (N=222), a tolterodine group (N=217), a tamsulosin group (N=215) and a combined treatment group (N=225). After 12-week treatment, it was found that, the combined treatment group had a remarkably lower incidence of urge urinary incontinence, 24-h urination frequency and night urination frequency and significantly improved IPSS and QOL. Chapple C et al.^13^ compared the effects of placebo and tolterodine on the basis of the application of α-receptor blocker and eventually drew out a similar conclusion. Solifenacin as M3-receptor blocker can significantly relieve the clinical symptoms of OAB patients compared to placebo and have been recommended as the first-line drug in the treatment of OAB.^14^

In this study, the subjective indexes such as IPSS, OABSS and QOL and the objective indexes such as PVR, Q_max_, daytime urination frequency, night urination frequency, urge urinary incontinence and urgent urination frequency in the two groups improved after treatment, and the differences had statistical significance (P<0.05). It indicated that, the clinical effect of tamsulosin in the treatment of prostatic hyperplasia was definite through inhibiting α_1A_ and α_1D_ receptors. The subjective and objective indexes in the observation group except PVR were significantly improved after treatment compared to the control group (P<0.05), suggesting solifenacin could inhibit the involuntary contraction of the detrusor, reduce detrusor overactivity, and lower bladder excitability, which is of great significance to the symptoms improvement of patients with BPH in combination with OAB. Solifenacin in combination with tamsulosin was more effective than tamsulosin alone in the treatment of BPH in combination with OAB. The difference of PVR before and after treatment of the two groups was not significantly difference (P>0.05), indicating tamsulosin was unable to improve bladder emptying degree and its efficacy was equivalent to tamsulosin.

M-receptor blocker associated adverse reactions, especially whether the incidence of acute urinary retention of BPH patients will increase, is concerned by all clinicians. Kaplan SA et al.^15^ enrolled 398 cases for a double-blind control study and found urinary retention occurred to 3% of patients who were treated with solifenacin in combination with tamsulosin. In the study of Yamaguchi O et al.^16^, patients who aged more than 45 years old, had no less than 200 mL of residual urine volume, no less than 5 mL/s of Q_max_, no less than 8 times of urination within hour and no less than 12 points of IPSS, and still had OAB after Harnal treatment were given solifenacin; it was found that, the urination frequency significantly reduced, and the overall tolerance was favorable. Wu SL. et al.^17^ compared the adverse reactions of patients who were treated by either solifenacin or tolterodine. The incidence of adverse reactions in the two groups was 11.7% and 23.5% respectively, and the incidence of mouth dryness which is the major adverse reaction was 5.8% and 10.4% respectively (P<0.05), suggesting solifenacin was more safe and effective in relieving OAB. A latest randomized, double-blind, parallel-controlled trial^18^ also suggested that, the risks of acute urinary retention was not increased when the patients were given the combined use of solifenacin and tamsulosin. In this study, the incidence of adverse reactions in the two groups suggested no remarkable difference, and there were no cases of acute urinary retention which needed catheterization, which were similar to the aforementioned research results and suggested the combined medication did not increase the incidence of adverse reactions.

## CONCLUSION

In conclusion, it is effective and safe to treat patients with BPH in combination with OAB with tamsulosin in combination with solifenacin, and its efficacy is better than solifenacin. As the sample size of this study was small, the comparison of adverse reactions between patients who are treated with combined medication or single medication remains to be investigated with studies with a large sample size.

### Authors’ Contribution

**HW & YHC:** Study design, data collection and analysis.

**YHC & HL:** Manuscript preparation, drafting and revising.

**WH & YHC:** Review and final approval of manuscript.
